# Role of antihypertensive medicines in prostate cancer: a systematic review

**DOI:** 10.1186/s12885-024-12218-5

**Published:** 2024-04-29

**Authors:** Chinonyerem O. Iheanacho, Okechukwu Harrison Enechukwu

**Affiliations:** 1 Department of Clinical Pharmacy and Public Health, Faculty of Pharmacy, University of Calabar, 540271 Calabar, Cross River State Nigeria; 2 Pharmacy Department, General Hospital Aboh, Aboh, Delta State Nigeria

**Keywords:** Prostate cancer, Hypertension, Antihypertensive medicines, Angiotensin receptor blockers, Calcium channel blockers, Renin-angiotensin inhibitors, Angiotensin-converting enzyme inhibitors

## Abstract

**Background:**

Hypertension is associated with the risk of prostate cancer (PCa) and its progression, however, it remains unclear whether antihypertensive medicines alter PCa risk or prognosis. This systematic review evaluated the role of calcium channel blockers (CCBs) and renin-angiotensin system (RAS) inhibitors in the risk and prognosis of PCa. This review was performed in line with PRISMA 2020 guidelines.

**Methods:**

Eligible studies comprised peer-reviewed observational studies which reported the role of CCBs and RAS inhibitors in PCa, had accessible full texts, and were written in English. Using a combination of keywords, 5 electronic bibliographic databases which included Web of Science, EMBASE, PubMed, Google Scholar and Scopus were searched.

**Results:**

A total of 1,346 studies were retrieved and 18 met the inclusion criteria. Thirteen studies reported reduced or no associated risk, improved prognosis, and survival with the use of RAS inhibitors. Studies on CCBs showed evidence of associated risk of PCa. Data extraction from retrieved studies focused on included study characteristics, setting, authors, year, outcomes of interest, and risk ratios. The quality assessment of included studies by the National Heart, Lung, and Blood Institute study assessment tools, showed that all studies had good quality.

**Conclusions:**

The use of RAS inhibitors was mostly associated with lower risks or improved prognosis of PCa. CCBs may also be associated with risks of PCa. This suggests that high-risk patients managed with CCBs should be actively monitored for PCa. However, there is need for further evidence from large-scale prospective, controlled cohort studies to determine any influence of CCBs on PCa.

**Supplementary Information:**

The online version contains supplementary material available at 10.1186/s12885-024-12218-5.

## Background

Cancers and cardiovascular diseases are the major causes of death in most countries, particularly the developed nations [1]. Prostate cancer (PCa) is the second most common cancer in males globally, and is associated with significant morbidity and mortality [2]. Aetiology of PCa involves environmental and genetic factors. PCa is also correlated with other factors such as metabolic syndrome, particularly hypertension [3–8]. Hypertension is a common cardiovascular risk factors and requires life-long therapy. However, clinical evidence has focused attention on the associated risk of PCa with long-term exposure to antihypertensive medicines [5, 9]. Given that hypertension is a suspected risk for PCa and its progression [5, 6], it remains unclear whether antihypertensive medicines reduce PCa risk or improve prognosis. However, as a result of their interference with cellular functions, commonly used antihypertensive medicines may pose a risk of PCa development or progression.

The relationship between antihypertensive medicines and PCa has been an area of increased interest. Most classes of drugs used in the management of hypertension (antihypertensive medicines) are notably effective at lowering blood pressure and they possess good safety profile. However, previous studies of diverse methodologies have suggested associations of the use of antihypertensive medicines and risk of PCa [9, 10]. The effects of these drugs on sympathetic nervous system and fluid homeostasis may have significant impact on PCa development and progression. Renin-angiotensin system (RAS) inhibitors are associated with a significant role in PCa, following their activity on the renin-angiotensin system. In vitro studies on PCa cell lines suggest autophagy-associated cell death and anti-metastatic effect of Angiotensin Receptor Blockers (ARB) and Angiotensin-converting enzyme inhibitors (ACEIs), and modulation of cellular processes central to the pathogenesis of PCa [11, 12]. Calcium channel blockers (CCBs) show autophagy effects on other type of cancers [13]. This class of medicines also show significant influence on the regulation of cell proliferation, differentiation and apoptosis possibly by its intracellular Ca^2+^ reduction [14]. In vitro tests showed that the L-type CCBs may cause a significant suppression of cell proliferation and androgen receptor-mediated gene expression in PCa, suggesting potential therapeutic effect on PCa cells [15]. This observation also suggests that the L-type calcium channel subunit may be a potential therapeutic target for PCa intervention [15]. Data from Chen et al., explains that this is achieved through the resulting suppression of androgen receptor transactivation, suppression of androgen-stimulating calcium influx, and suppression of cell growth in PCa cells, from the blockade of L-type channel’s activities [16]. Therefore, altered risk of PCa is suggested in users of CCBs and RAS inhibitors. This also suggests potential positive effects in PCa management. However, a clinical evidence reported higher associated risk of PCa with the use of calcium channel blockers (CCBs) (RR 1.10, 95% CI 1.04–1.16), unlike ARBs (RR1.09, 95% CI 0.97–1.21) and ACEIs (RR1.07, 95% CI 0.96–1.20) [9]. CCBs and antihypertensive medicines that target the renin-angiotensin system were selected for this review as a result of their very wide use, since they are first line antihypertensive drug classes [17, 18], and possess potential evidence of association with PCa [9].

The role of antihypertensive medicines in risk and prognosis of PCa is a long term debate. Therefore, adequate understanding of the role of antihypertensive medicines on the risks of PCa is relevant for improving knowledge of the predisposing pathophysiological changes and mechanisms of PCa aetiology and progression, which is relevant for improved patient care. There is paucity of systematic evidence on the associated role of these medicines in the prognosis of PCa. Although a previous systematic evidence focused on risk of RAS inhibitors and CCBs on PCa [9], this systematic review includes more recent evidence to further enhance knowledge on the associated risks. It also includes a systematic evidence of the associated effects of the medicines on PCa prognosis, which is scarcely available. Therefore, using available evidence, this review evaluated role of CCBs and RAS inhibitors in the risk of developing PCa and the prognosis of PCa. This knowledge will enhance clinical practice and improve patient outcomes.

## Main text

### Study design

The systematic review was performed in line with the Preferred Reporting Items for Systematic Reviews and Meta-Analysis (PRISMA) guidelines 2020 [19]. Systematic review of all eligible articles was conducted. The PRISMA 2020 statement comprises a checklist of 27 essential items to ensure reporting transparency. See Supplementary 1.

### Outcomes of interest

The primary outcome of interest in this study was the associated risk of PCa with the use of antihypertensive medicines that target the renin-angiotensin system (RAS) and those that block the calcium channels (CCBs). The secondary outcome measure was the associated prognosis of PCa in the use of the specified antihypertensive medicines.

### Participants

No limits were observed for the social status, age or race of participants, and all participants were drawn across the international borders. The target population for this review were adults with hypertension who were managed with RAS inhibitors and CCB classes of antihypertensive medicines. Also, this cohort of participants where only those who had information on prostate cancer incidence and/or prognosis, limiting the target to men.

Antihypertensive medicines were defined as any class of medicines used to manage high blood pressure. Persons with varied degree of hypertension and varied duration of treatment with the classes of antihypertensive medicines were targeted. In the context of this study, risk of PCa is defined as the associated ability of the studied classes of drugs to cause the occurrence of PCa in users of the drugs, while the disease prognosis refers to the disease outcomes associated with the use of any class of the studied drugs.

### Eligibility criteria

Studies were included in the review if they were on the risk and prognosis of PCa with the use of RAS inhibitors or CCBs, peer-reviewed, original, conducted in any continent, observational (cohort, case-control) and published in English language between January 1, 2000 and November 31, 2022. Aside searching peer reviewed literature, the authors also performed a search of publications from key institutions and other grey literature from government and organisations’ websites. As a result of unavailability of logistical and financial capacity, the authors could not retrieve or translate literature published in languages other than English, hence they were excluded.

### Search strategy

Comprehensive search of peer-reviewed articles in 5 databases: Web of Science, PubMed, Google Scholar, Embase, and Scopus was done using appropriate search terms, Boolean operators (“AND”, “OR” and “NOT”) and subject heading truncations ( *). These were modified to ensure adherence to the specifications of each of the searched databases. The bibliographical references of all eligible articles were also searched to include any previously omitted or additional relevant articles. The following keywords were used in various combinations: prostate, cancer, carcinoma of the prostate, antihypertensive, medicines, amlodipine, nifedipine, captopril, lisinopril, calcium channel blockers, angiotensin receptor blockers, renin-angiotensin-aldosterone system, angiotensin converting enzyme inhibitors.

### Study selection

All studies retrieved from the included databases were screened and duplicates were removed. The titles and abstracts were further screened to determine relevance. Furthermore, eligibility of the remaining studies were determined after reading of full texts, and this led to the retaining of studies that met the inclusion criteria. The entire screening process was performed independently by the two researchers and a consensus was reached for any disagreement. The study selection is presented in a PRISMA flowchart [20].

### Data extraction and synthesis

The extracted data were study authors, study design, year of publication, study settings, study size, risk quantification (risk ratio RR, hazard ratio HR, odds ratio OR and the 95% CI), outcomes of interest. Data extraction was done by COI, and OHE, and independently reviewed by the first author. Areas of conflict where resolved through re-evaluation of the article and consultation of relevant literatures. Subsequently, both authors studied the bibliographical references of all eligible studies to identify other relevant studies. Narrative synthesis was used to synthesise results. This method was adopted to avoid bias associated with other methods of synthesis. Funnel plot and heterogeneity test were performed to provide a visual aid for detecting bias or systematic heterogeneity. Results were categorised into 4 sections which include, search results, characteristics of included studies, results of statistical analyses and summary of findings.

### Quality of selected studies

Quality assessment was based on the evaluation of the methodological quality of included studies. The study assessment tool of the National Heart, Lung, and Blood Institute of the National Institutes of Health (NIH) for quality assessment of Observational Cohort and Cross-Sectional Studies, was used to assess the quality of retained cohort studies [21]. The included case-control studies where assessed for quality by using the quality assessment tool of the National Heart, Lung, and Blood Institute of the National Institutes of Health (NIH) for quality assessment of case-control studies [21]. Available evidence suggests that the NIH quality assessment tool is efficient in the determination of risk of bias in cohort studies [22, 23]. The NIH quality assessment tool for cohort and observational studies uses a checklist that measures 14 criteria for assessing the external validity and internal validity. The external validity is associated with potential selection bias, while the internal validity is related to confounding bias and potential measurement biases of the retained studies. All included studies were termed good, fair or poor according to the associated score. A study was termed ‘good’ if it reached 10–14 points, ‘fair’ if it reached 5–9 points and ‘poor’ if it was ≤ 4 points. This is shown in supplementary 2. Meanwhile, the NIH quality assessment tool for case-control studies comprises a checklist of 12 items that measure the validity of case-control studies, and quality was also rated as good, fair or poor. This is shown in supplementary 3. Internal validity for case-control studies refers to the extent to which the associations between exposure and the reported disease in the study can truly be attributed to the exposure being evaluated rather than to errors associated with the study such as, flaws in the design or conduct of the study [21]. Higher scores implied lower risk of bias [21].

## Results

### Search results

**Fig. 1:**
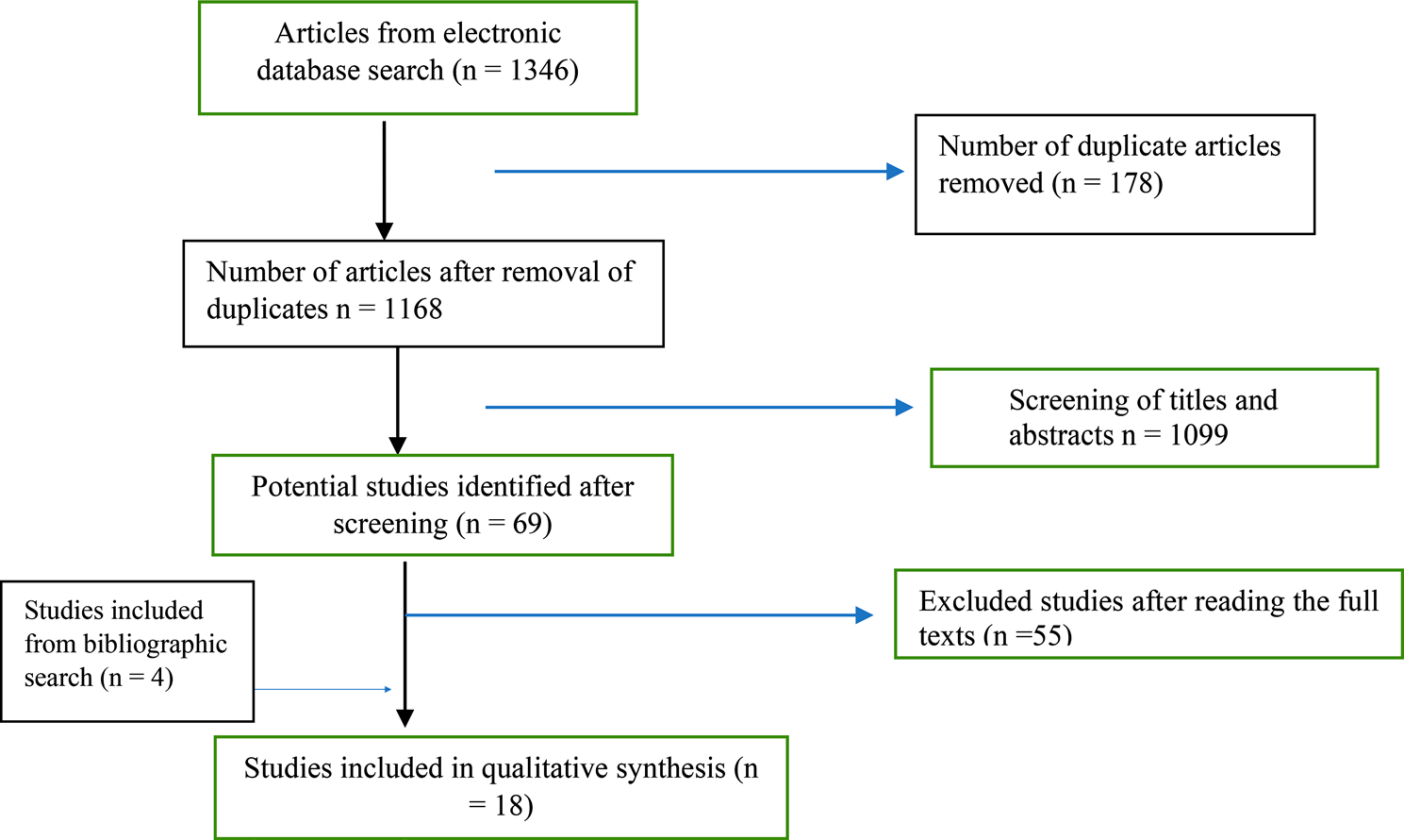
**PRISMA flow diagram of study selection**

A total of 1,346 studies were retrieved from 5 databases. A total of 178 duplicates were removed, and this led to the retention of 1168 studies which were further screened. After screening of the titles and abstracts, a total of 1,099 studies were excluded. Furthermore, 69 studies were retrieved and full-texts assessed for eligibility, and this resulted in the exclusion of 55 studies. Therefore, only 14 studies met the inclusion criteria. A search of the reference lists of all retained studies identified 4 additional relevant studies, and this resulted in a total of 18 included studies. Study selection process is shown in the PRISMA flow diagram (Fig. 1).

### Characteristics of included studies

Table 1 shows a summary of the studies included in this review. A total of 18 studies were included in the review. Four studies were conducted in Finland [10, 24–26], 6 in US [27–32], 1 in UK [33], 1 in Denmark [34], 1 in Poland [35], 1 in Sweden [36], 1 in Israel [37], 1 in China [38], 1 in Canada [39] and 1 in Taiwan [3]. Eleven were population-based cohort studies [10, 25–31, 33–35], while 7 were case-control studies [3, 24, 32, 36–39]. The participants’ follow-up ranged from 3 to 20 years and all the studies but 1, were retrospective. Nine studies reported on the relationship between PCa risk or prognosis with the use of CCBs. Nine studies also reported relationship of PCa risks and prognosis with ACEIs. Eight studies reported associations of ARBs and PCa risks and prognosis. See Supplementary 3 Table. All included studies were published between 2001 and 2021. Findings from the quality assessment of included studies showed that all studies were of good quality.

### Results of statistical analyses

The results of heterogeneity test of the included studies showed a heterogeneity of 85%, using 95% confidence interval. This implies that there was considerable heterogeneity, hence a meta-analysis could not be performed. Heterogeneity: Chi^2^ = 106.90, df = 16 (*P* < 0.00001); 1^2^ = 85%. The test for overall effect: Z– 7.34 (*P* < 0.00001). See supplementary 4 for the funnel plot, forest plot and heterogeneity test.

### Summary of findings

In general, a study noted that the use of antihypertensive drugs was associated with a similar and slightly elevated risk of PCa (HR = 1.16, 95% CI = 1.11–1.22) and metastatic PCa (HR = 1.36, 95% CI = 1.14–1.62) [10]. Another study also observed a similar association for PCa risk across the studied antihypertensive medicines (OR 1.16; 95% CI, 1.12–1.21) [24]. Studies by Kemppainen et al., and Fitzpatrick et al., reported absence of associated difference in risk of PCa and advanced PCa with the use of any specific class of antihypertensive medicine (OR 1.08, 95% CI 0.98–1.18) and (HR: 0.7, 95% CI: 0.5–0.9), respectively [24, 27], although Fitzpatrick et al. also reported an inverse association between PCa and use of CCBs [27]. Meanwhile, the study by Fitzpatrick et al. was limited by a smaller sample size, but presented information on the cohort’s drug use and blood pressure levels [27], while Kemppainen et al. had a case-control design, a large sample size and a long duration of follow-up [24]. Two matched case-control studies with large sample sizes and long follow-ups also reported no clear or associated risk between long-term use of antihypertensive medicines and PCa [3, 36]. Meanwhile, the use of antihypertensive medicines was associated with elevated risk of PCa specific death in two population-based cohort studies [25, 26] and risk of initiation of androgen deprivation therapy (ADT) [26]. It is important to note that the users of antihypertensive drugs may likely have other comorbidities and are at increased risk of death in general as compared to non-users, which may possibly influence the reported PCa-specific mortality. Also, data for reimbursement for drug purchase was extracted for the study by Silltari et al., with no clear information on the actual use of the drugs, posing a limitation to the study [26]. In contrast, lower risk [26] and inverse association between risk of PCa and antihypertensive medicine use [24] were observed by other studies. Confounders such as behavioural and other health status with potential shared risks with PCa may have influenced findings in some studies, as lack of data availability prevented relevant statistical adjustments of these risks in the studies. Risk quantifications and other study characteristics are shown in Table 1.

Among 9 studies that reported the relationship between PCa risk or prognosis, with the use of CCBs, 4 associated the use of CCBs with increased risk of PCa [24, 28, 37, 38]. Among these, studies with large sample sizes and longer follow-ups reported slightly elevated risk of PCa with the use of CCBs (OR 1.16; 95% CI, 1.12–1.21) and 1.14 (95% CI: 0.95–1.36), respectively [24, 38]. The reported case-control study by Kemppainen et al., had a 24,657 cohort and a 7-year follow-up duration [24]. Similarly, the study by Kao et al., was a population nested case-control study of a large cohort comprising 23,666 participants and a 5-year follow-up duration [38]. Meanwhile, studies that had smaller sample sizes and shorter follow-up reported significant PCa risk association with the use of CCBs OR: 95% CI: 0.55 (0.31–0.97) and (OR 1.10, 95% CI: 1.02–1.18), respectively [28, 37], and the risk increased with duration of use (association for PCa increased by 27% for every 10-year increment of CCB use (OR 1.27, 95% CI 1.04–1.56).) [37]. In these studies, Debes et al., and Rotshild et al., observed a small cohort of 1,362 in a 2-year follow-up duration, and 4,346 in a 5.3 years follow-up duration, respectively [28, 37], It is pertinent to note that these studies were limited by several factors. Most importantly, they did not account for possible confounders such as environmental exposures, dietary and other lifestyle-related risk factors. Also, one study did not have a large sample size but had a long follow-up duration [37]. Four other studies associated use of CCBs with reduced risk, reduced aggressiveness or no associated PCa risk [(1–5 year vs. non-users HR = 0.99, 95% CI = 0.32–3.05; >5 year vs. non-user HR = 0.88, 95% CI = 0.34–2.26), (HR: 0.7, 95% CI: 0.5–0.9), (Gleason scores ≥ 7: adjusted OR = 0.64; 95% CI: 0.44–0.950 and 0.98 (CI, 0.88–1.08)], respectively [3, 27, 32, 39]. The study that observed CCB-associated reduced PCa aggressiveness had a case-control, but had a smaller sample size of 1,747 and a 3-year follow-up [32]. On the contrary, study by Poch et al. reported that CCBs were not associated with the outcomes of PCa, including PCa aggressiveness at diagnosis, progression-free survival or overall survival (median range PSA for non-CCB users; 5.44 (0.23–90), CCB users: 5 (1.50–29). *p* = 0.97; aggressiveness: Gleason sum (*p* = 0.61), Tumor T stage (*p* = 0.88), Tumor aggressiveness (*p* = 0.88).), but did not report on the risks of PCa with the use of CCBs [30]. This study was limited by a smaller sample size (875) and a shorter duration of follow-up (2 years). Similarly, one study observed that exposure to CCBs was associated with lower relative risks for increased Gleason scores and T2F positive PCa (Gleason scores ≥ 7: adjusted OR = 0.64; 95% CI: 0.44–0.95) [32]. Perron et al., also found that use of CCBs was not associated with PCa risks (0.98 (CI, 0.88–1.08)) [39]. Although the sample size was large (13,326) and there was control for detection bias, several confounders such as dietary exposures were not accounted for. See Table 1 for risk quantifications and other study characteristics.

Out of 9 studies that reported on ACEIs and risks of PCa, 7 associated ACEIs with improved prognosis, reduced or no associated risk with PCa [3, 27, 29, 34–36, 39]. Conversely, Silatri et al. and Kemppainen et al. reported an associated slightly elevated risk of PCa with use of ACEIs (HR = 1.10, 95% CI = 1.01–1.19 for PCa; OR 1.16; 95% CI, 1.12–1.21), respectively [10, 24]. Although these studies [10, 24] had long follow-up durations (20 years and 7 years, respectively) and large sample sizes (80,456 and 24,657, respectively), they did not account for the impact of relevant potential confounders on the relationship between ACEIs use and the risk of PCa. During their study of a 48,389 cohort for an 8-year period, Rodriguez et al., observed that the use of ACEIs were associated with lower PCa risk than other antihypertensive drugs when adjusted for age and race (OR = 0.10) [29]. More specifically, lower risk for PCa was associated with the use of captopril (RR = 0.7 (95% CI: 0.4–1.2)) in a nested case-control study of 243,331 cohort within a 4-year follow-up period [36]. Fitzpatrick et al., also observed an inverse association between exposure to ACEIs and PCa risks (HR: 0.7, 95% CI: 0.5–0.9) in a smaller sample sized (2,442) cohort study, with possible influence of confounders [27]. The cohort had a long follow-up of 7 years. In contrast, the study by Perron et al. reported absence of association between PCa risk and use of ACEIs (0.98 (CI, 0.88–1.08) in a cohort of 13,326, but as earlier noted, had a smaller sample size however, its inclusion of a control for relevant confounders with a long duration of follow-up enhanced the strength of the study [39]. Similarly, no association was observed between ACEIs and the risk of developing PCa in a matched case-control study of a 402,215 cohort by Pai et al., (1–5 year vs. non-users HR = 0.99, 95% CI = 0.32–3.05; >5 year vs. non-user sHR = 0.88, 95% CI = 0.34–2.26.) [3]. The study which also had a large sample size and a long follow-up period of 9 years matched cases with control for potential confounders. Findings by Friis et al., also suggest the absence of association between use of ACEIs and the risks of PCa incidence (HR: 1.01 (95% CI, 0.93–1.09; comparable to non-users) [34]. The study had a large sample size of 17,897 and a mean follow-up duration of 3.7 years, but did not include a case-control. Meanwhile, WIlk et al., observed a positive association between PCa and ACEI use [35]. In their cohort of 93 participants, the researchers reported improved PCa prognosis in the use of ACEIs observed as a longer time to treatment failure (TTF) (HR, 0.61; 95% CI 0.4–0.94; *p* = 0.02.). The small sample size and absence of controls were major draw backs of this study. Risks quantification is shown in Table 1.

Eight studies reported associations between ARBs and PCa risks and prognosis, among which 6 observed improved prognosis or no associated risk with PCa [3, 25–27, 31, 35], while two studies reported associations between ARBs and elevated PCa risk [24, 33]. Among the studies that observed an elevated risk of PCa with ARBs, one study reported a weak association with elevated PCa risk (1.10, 1.00 to 1.20, *p* = 0.04) [33]. The observed evidence of an increase in the risk of PCa among users of ARBs was reported to be small in absolute terms and the observed risk was not associated with the duration of ARB use (*p* > 0.15) and as such, may have resulted from other risk factors for PCa [33]. The major limitations of this study were the absence of study control with increased risk of confounder bias however, it had a long follow-up period of 4.6 years and studied a large sample comprising a cohort of 20,203. Specifically, angiotensin system inhibitors were linked to improved prognosis of PCa in persons with castration-resistant cancer on abiraterone in a small sized cohort study of 93 participants, where relevant confounders were not controlled [35]. The observed statistical significance remained after adjustment for known oncological factors (HR, 0.57; 95% CI, 0.34–0.98; *p* = 0.04). Median TTF of 12.2 months versus 5.8 months in men who did not receive ASI.) [35].Similarly, in their study of a cohort comprising 14,422 participants, Santala et al., observed that angiotensin II type 1 receptor blockers were associated with improved survival (HR: 0.43, 95% CI: 0.26–0.72 and HR: 0.60, 95% CI 0.37–0.97 for pre- and post-diagnostic use) and lowered risk of commencing androgen deprivation therapy (ADT) (HR: 0.81 CI:0.71–0.92), but findings were limited by lack of a relevant control [25]. ARBs were also associated with anti-cancer effects and improved PCa prognosis (0.81 (0.67–0.99) in a population-based cohort study of 8,253, and a median follow–up duration of 7.6 years [26]. Likewise, ARBs were also observed to be minimally but significantly associated with a reduction in incidence of clinically detected PCa, but not associated with degree of differentiation (HR = 0.91; *P* = 0.049) in another study [31]. This study had a very large sample size of 543,824 participants but was limited by the non-inclusion of a control and non-adjustment for potential confounders. Risk quantification of the included studies is shown in Table 1.

**Table 1:** Characteristics of selected studies

Authors, Year Country	Study design	Study characteristics	Outcomes	Risk quantifications	Quality assessment
Silatri et al., 2018 [10] Finland	Retrospective (population-based cohort)	Sample size: 80,456 Follow-up: 20 years	Antihypertensive medications, specifically ACEIs were associated with slightly increased PCa risk	Small excess increased risk HR = 1.10, 95% CI = 1.01–1.19 for PCa	12
Siltari et al., 2020 [26] Finland	Retrospective (population-based cohort)	Sample size: 8,253 Follow-up: 7.6 years (medians)	Antihypertensive drug use overall was associated with an increased risk of PCa specific death. However, anticancer effects and improved prognosis of PCa was observed for renin-angiotensin type 1 receptor blockers	(Pre-PCa: 1.21 (1.04–1.4), Post-PCa: 1.2 (1.02–1.41)) 0.81 (0.67–0.99)	11
Santala et al. 2019 [25] Finland	Retrospective cohort	Sample size: 14,422	Only ARBs were associated with improved survival and reduced risk of initiating androgen deprivation therapy (ADT) after radical prostatectomy. Increased risk of initiating ADT was reported for other antihypertensive medicines	Decreased risk of PCa death (HR: 0.43, 95% CI: 0.26–0.72 and HR: 0.60, 95% CI 0.37–0.97 for pre- and post-diagnostic use). Reduced risk of commencing ADT (HR: 0.81 CI:0.71–0.92).	12
Bhaskaran et al., 2012 [33] Uk	Retrospective cohort	Sample size: 20,203 Follow-up: 4.6 years	There was some evidence of slightly increased risk of PCa in ARB users, but lack of association with duration of treatment meant that non-causal explanations could not be excluded.	From 1.10, 1.00 to 1.20, *p* = 0.04; which in absolute terms corresponded to an estimated 1.1 extra cases, per 1000 person years of follow-up among those with the highest baseline risk. No association with duration: *P* > 0.15.	13
Wilk et al., 2021 [35] Poland	Retrospective (cohort)	Sample size: 93	Renin angiotensin system inhibitors linked to improved PCa outcomes	Longer time to treatment failure (TTF): HR, 0.61; 95% CI 0.4–0.94; *p* = 0.02. Statistical significance remained after adjustment for well-known oncological factors (HR, 0.57; 95% CI, 0.34–0.98; *p* = 0.04). Median TTF of 12.2 months versus 5.8 months in men who did not receive ASI.	13
Rotshild et al., 2019 [37] Israel	Retrospective (Nested case-control study	Sample size: 4,346 Follow-up: 5.3 years	CCBs was significantly associated with elevated risk of PCa, and the risk increased with duration of use.	Increase in risk for PCa (OR 1.10, 95% CI: 1.02–1.18). Association for PCa increased by 27% for every 10-year increment of CCB use (OR 1.27, 95% CI 1.04–1.56).	10
Geybels et al. 2017 [32] USA	Retrospective (population-based case-control)	Sample size: 1,747 (control = 1,635) Follow-up: 3 years	CCBs was relatively associated with lower risks for higher Gleason score and *T2F* positive PCa	Gleason scores ≥ 7: adjusted OR = 0.64; 95% CI: 0.44–0.95.	10
Ronquist et al. 2004 [36] Sweden	Retrospective (nested case-control)	Sample size: 243,331 (cases: 1,013) Follow-up: 4 years	Lower risk of PCa associated with the use of captopril	Relative risk of 0.7 (95% CI: 0.4–1.2)	11
Fitzpatrick et al. 2001 [27] USA	Retrospective (cohort)	Sample size: 2,442 Follow-up: 7 years	Inverse association between PCa and use of antihypertensive medicines (ACEIs, ARBs and CCBs). There was also no difference between use of the specific classes of antihypertensive medication and associated PCa risk.	HR: 0.7, 95% CI: 0.5–0.9	13
Debes et al. 2004 [28] USA	Prospective (cohort)	Sample size: 1,362 Follow-up: 2 years	Daily use of CCBs was associated with risk of PCa, and it varied by family history of PCa.	The risk (OR: 95% CI: 0.55 (0.31–0.97), stratified by family history, the risk was 0.45 (0.23–0.88) in men without a family history and 2.64 (0.82–8.47) in men with a family history (*P* = 0.006).	11
Perron et al. 2004 [39] Canada	Retrospective (matched case-control)	Sample size: 13,326	PCa was not associated with the use of CCBs and ACEIs.	0.98 (CI, 0.88–1.08)	11
Kemppainen et al. 2011 [24] Finland	Retrospective (case-control)	Sample size: 24,657 Follow-up: 7years	ARBs, ACEIs and CCBs were associated with similar and marginally elevated risks of PCa	Marginally elevated risk (OR 1.16; 95% CI, 1.12–1.21). Risk of advanced prostate cancer did not differ from the nonusers (OR 1.08, 95% CI 0.98–1.18)	10
Poch et al. 2013 [30] USA	Retrospective (cohort)	Sample size: 875 Follow-up: 2 years	CCBs were not associated with PSA values at diagnosis and PCa aggressiveness	Median range PSA: Non-CCB users; 5.44 (0.23–90), CCB users: 5 (1.50–29). *P* = 0.97 Aggressiveness: Gleason sum (*p* = 0.61), Tumor T stage (*p* = 0.88), Tumor aggressiveness (*p* = 0.88).	12
Kao et al. 2018 [38] China	Retrospective (population-based case-control)	Sample size: 23,666 Follow-up: 5 years	PCa risk was slightly associated with CCBs use.	1.14 (95% CI: 0.95–1.36).	10
Pai et al. 2015 [3] Taiwan	Retrospective (matched case-control cohort)	Sample size: 402,215 Follow-up: 9 years	Long-term use of antihypertensive medicines was not associated with risk of developing PCa.	1–5 year vs. non-users HR = 0.99, 95% CI = 0.32–3.05; >5 year vs. non-user sHR = 0.88, 95% CI = 0.34–2.26.	11
Rao et al. 2013 [31] USA	Retrospective (cohort)	Sample size: 543,824	ARBs did not increase the risk of incident PCa. There was small but significantly associated reduction in the incidence of PCa, in the use of ARBs. ARBs were not associated with degree of PCa differentiation.	Post weighting, the rates of PrCA in treated (ARBs) and not-treated groups were 506 (1.5%) and 8,269 (1.6%), respectively; representing a hazard ratio of 0.91, *P* = 0.049.	11
Friis et al. 2001 [34] Denmark	Retrospective cohort	Sample size: 17,897 Follow-up: 3.7 years (mean)	ACEIs was not associated with protective effects against incidence of cancer	HR: 1.01 (95% CI, 0.93–1.09) comparable to non-users	12
Rodriguez et al. 2009 [29] USA	Retrospective cohort	Sample size: 48,389 Follow-up: 8 years	ACEI was associated with an approximately 10% lower risk for all PCa in models adjusted for age and race. However, strong associations with risk of all PCa were lost after adjustment for history of heart disease.		13

## Discussion

Although few studies in this review found an associated risk of PCa across the classes of antihypertensive medicines, the main findings suggest positive or no associations between risk or worsened prognosis of PCa with the use of ACE inhibitors and ARBs. Conversely, exposure to CCB appeared to be associated with the risk of PCa. Moreover, the presence of potential confounders including hypertension may have strongly contributed to evidence levels.

Association of antihypertensive drugs with the risk of PCa was observed at varied levels across all classes, suggesting that PCa is probably associated with a systemic difference between medication users and non-medication users. In their analysis, Fitzpatrick et al., buttressed the impact of untreated hypertension on PCa risks in comparison to treated hypertensive and normotensive cases [27]. Therefore, the observed risk for PCa across different groups of antihypertensive medicines may also suggest correlation of PCa with hypertension rather than the antihypertensive medicines [10]. Although Fitzpatrick et al. [27]., found no association between blood pressure measures and incident PCa, previous studies have noted that hypertension is a risk factor for PCa, and increased risk of death from PCa [3, 4]. This risk may be associated with the involvement of the sympathetic nervous system. More so, several confounders, including current illness and other metabolic factors may explain the inverse association reported in other studies. Although several factors may be responsible for this observation, varied clinical and in vitro studies have demonstrated the associated role of various classes of antihypertensive medicines on the risks and prognosis of PCa [12, 15, 16, 40, 41].

Meanwhile, the possible association of the use of some drugs which have positively enhanced quality of life, life expectancy and CVD outcomes, with the alteration of the risk of PCa in long term use, is of important concern. Although the relationship between CCB usage and the risk of PCa lacked consistent evidence in clinical research, most studies in this review associated increased risk of PCa with the use of CCBs, meanwhile in vitro studies suggest otherwise. In vitro analysis showed that the L-type CCBs such as nifedipine significantly suppress cell proliferation and androgen receptor-mediated gene expression in PCa, suggesting potential therapeutic effect on PCa cells [15]. This suggests that the L-type calcium channel subunit (cav3.2) may be a potential therapeutic target for PCa intervention [15]. According to data from a previous study, this is achieved through the suppression of androgen receptor transactivation, suppression of androgen-stimulating calcium influx, and suppression of cell growth in PCa cells by the blockade of L-type calcium channel’s activities [16]. This in vitro data corroborates the findings by Debes, et al. which suggests an inverse association between PCa and the use of CCB, although results varied according to a family history of PCa [28]. A previous meta-analysis of 9 studies also found no significant association between CCBs and incidence of PCa, and suggested that CCBs may be protective of PCa in older men [40]. On the contrary, in a meta-analysis of 21 observational studies from varying classes of antihypertensives, Cao et al., associated increased risk of PCa with the use of CCBs, but not with other antihypertensive medicines [9]. The limitations of individual studies and heterogeneity of included studies may have resulted in these significant differences in findings. Results of a large population-based study also showed a modest but significant duration of use-dependent elevated risk of PCa among CCB users [37]. Given that calcium channel blockers target calcium channels which regulate calcium homeostasis, they interfere with cellular processes that are necessary in cancer, such as proliferation and apoptosis [42]. An increase in calcium channel activity may be correlated with higher cellular proliferation and cancer growth.

Findings suggest associated anticancer effects, lower risks, or improved prognosis of PCa with the use of antihypertensive medicines that target the RAS. However, Rodriguez et al. observed that the associations were lost when adjusted for history of heart disease [29], which suggests the influence of confounder of concurrent illness. Therefore, confounders are important factors to be critically assessed. Meanwhile, RAS inhibitors have been previously noted to possess beneficial effects in primary and metastatic tumors [43]. The activity of this class of medicines on the RAS appears to be responsible for their role in PCa. In vitro studies on PCa cell lines suggest autophagy-associated cell death and anti-metastatic effect of ARBs and ACEIs, and modulation of cellular processes central to the pathogenesis of PCa [11, 12]. Consistent with findings in this review, Cao et al., reported no significant association between ACEIs and risk of PCa, in a meta-analysis of 10 studies [9]. Similarly, a pool-analysis of 5 studies also showed no significant relationship between ARB use and the risk of PCa [9]. In their studies, Siltari et al., suggested that the use of ARBs was associated with improved survival of PCa patients [26], while Wilk et al., reported longer time to treatment failure in ARBs use, compared to other classes of antihypertensive drugs, in patients with castration-resistant PCa [35]. These suggest that RAS inhibitors may influence the modification of gene expression, thus inhibiting proliferation and invasion of cancer cells, thereby limiting endothelial cell migration and angiogenesis [11, 44]. As demonstrated in previous analysis, the activities of ACEIs interfere with on vascular endothelial growth factor to lower its levels in tumors, thereby preventing the formation of masses from blood vessels, which results in nutrient deprivation and subsequent hindered growth [45]. Losartan is observed to exhibit lethal effects on PCa cells, largely reducing cell survival, in vitro [41]. It also induces apoptosis of other cancer cells [46]. This also corroborates findings in a clinical study where statistically significant reduction in the incidence of clinically detected PCa in patients who received ARB was observed [31]. Another study also associated ACEIs and ARBs with decreased risk and improved outcomes of other cancers [47]. Similarly, a meta-analysis by Mao et al., comprising a total of 20,267 patients from nine cohort studies also found that use of RAS inhibitors may be associated with a decreased risk of PCa [48]. The study had no evidence of significant publication bias. This suggests that ARBs may possess prophylactic and therapeutic effects in PCa, given that proliferation of prostate cells is mediated by angiotensin II, and ARBs through suppression of MAPK or STAT3 phosphorylation [49].

Available evidence suggests that CCBs may be associated with PCa, and as such, findings of this study will aid in the provision of tailored drug therapy to males with hypertension, particularly those at high risk of developing PCa. For instance, findings suggest that high-risk patients managed with CCBs may require active or routine monitoring for PCa. This systematic review also highlights the gap in available evidence on the role of CCBs in PCa, thereby showing the need for more research which should examine the role of CCBs in the risk of PCa, prognosis, and survival. This identified gap will inform future research efforts. Evidence from the review also reinforces that RAS inhibitors are not associated with increased risk or worsened prognosis of PCa, This finding further supports the position statement of the US Food and Drug Administration, which affirms that there is no increase in the risk of cancer with the use of ARBs [50]. Therefore, the use of RAS inhibitors should not be discouraged in persons with identified needs irrespective of their PCa risks or status.

This study comprehensively searched existing literature on the role of CCBs and RAS inhibitors on PCa. The study involved a systematic and transparent method for reproducible data synthesis. Nonetheless, some limitations are associated with the study. Literature search was limited to 5 databases, this may have excluded potentially relevant studies. All included studies but one, were retrospective, and are associated with the inherent risks of bias of observational studies which include the risk of confounders. Furthermore, studies published in languages other than English were excluded, and this may have led to the loss of key findings from literatures by non-English-speaking researchers. Also, hypertension and metabolic syndrome being risks for PCa may have been major confounders in the studies. Further, it should be noted that persons with hypertension or other confounding factors most probably are actively and/or closely monitored by medical personnel compared to the control population in epidemiological settings which might lead to more frequent testing of other diseases, such as PSA testing activity. This may have resulted in more frequent incident of PCa observed among antihypertensive drug users in the reported studies. It is also important to note that most of the evidence in this study are based solely on epidemiological findings rather than clinical evidence.

## Conclusion

The use of RAS inhibitors was mostly associated with lower risks or improved prognosis of PCa. Findings also show that CCBs may be associated with risks of PCa. This suggests that high-risk patients managed with CCBs may require active monitoring for PCa. However, there is need for further evidence from large-scale prospective, controlled cohort studies to determine any influence of CCBs on PCa.

### Electronic supplementary material

Below is the link to the electronic supplementary material.

Supplementary Material 1

Supplementary Material 2

Supplementary Material 3

Supplementary Material 4

## Data Availability

Data from quality assessment of included studies, and results of heterogeneity test, funnel and forest plots are included as supplementary material (supplementary files 1 to 4).

## Declarations

### Review protocol

Not available.
