# Supplementary figures and images for: Role of antihypertensive medicines in prostate cancer: a systematic review
Source: BMC Cancer. 2024 Apr 29;24:542. doi: 10.1186/s12885-024-12218-5 (PMC11059764; doi:10.1186/s12885-024-12218-5)

FUNNEL PLOT

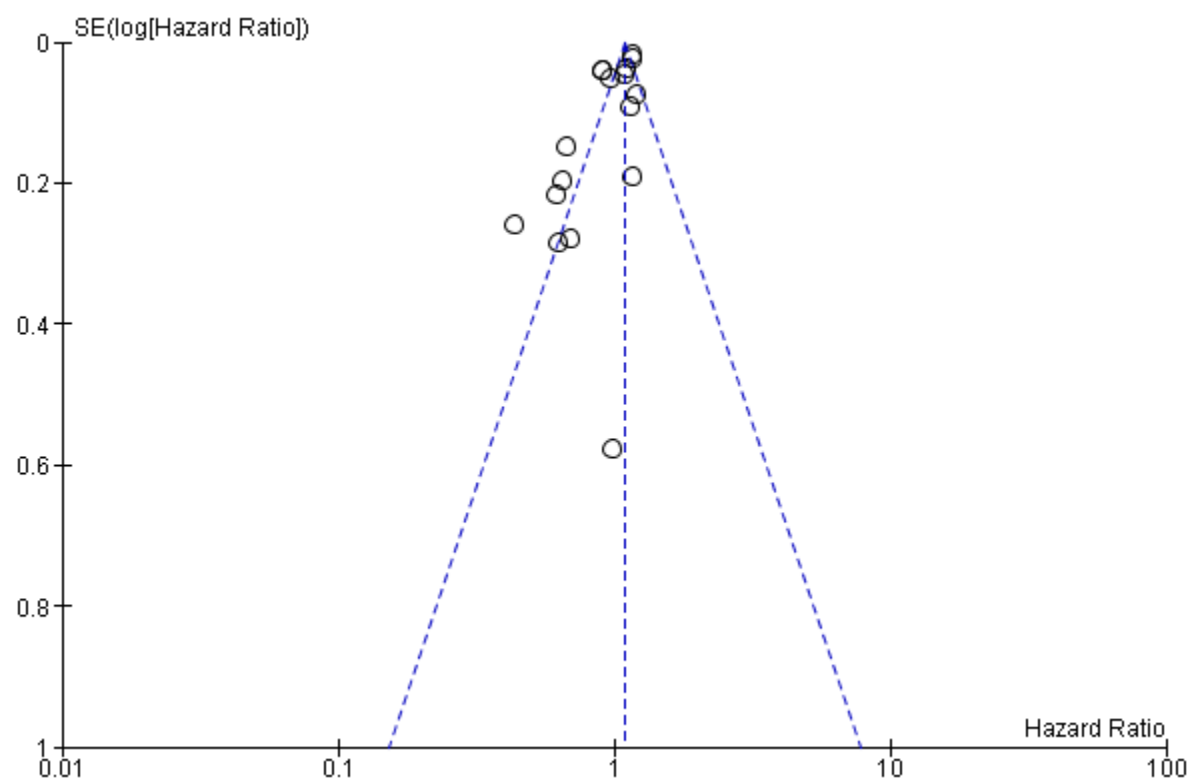

## Forest plot

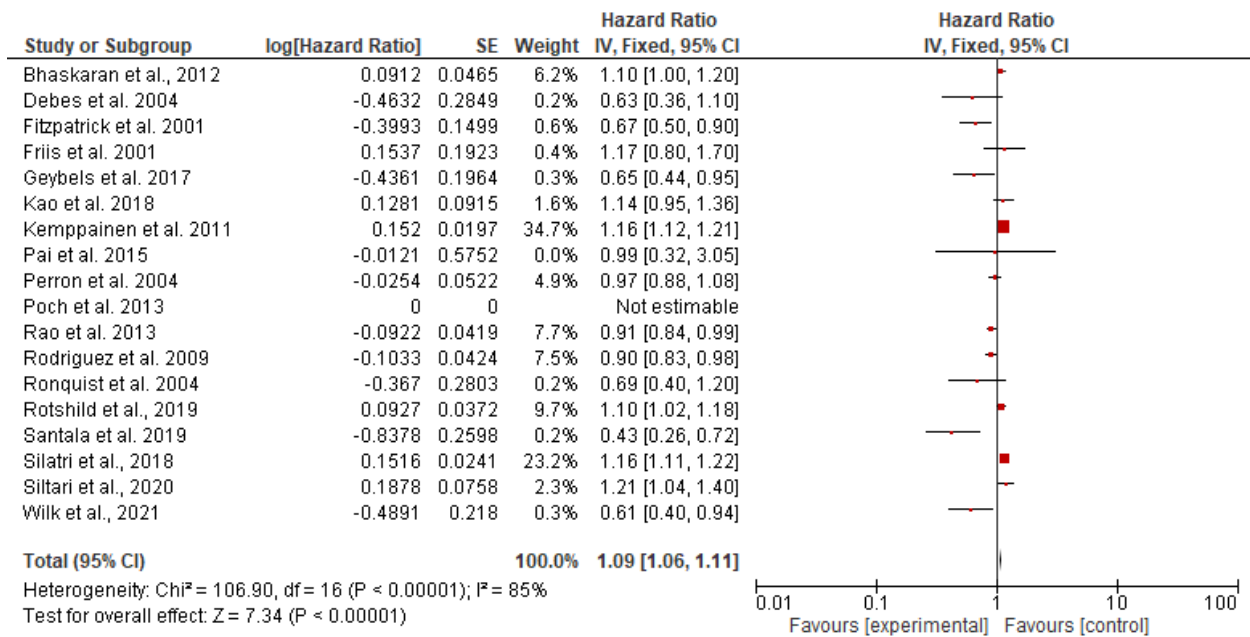

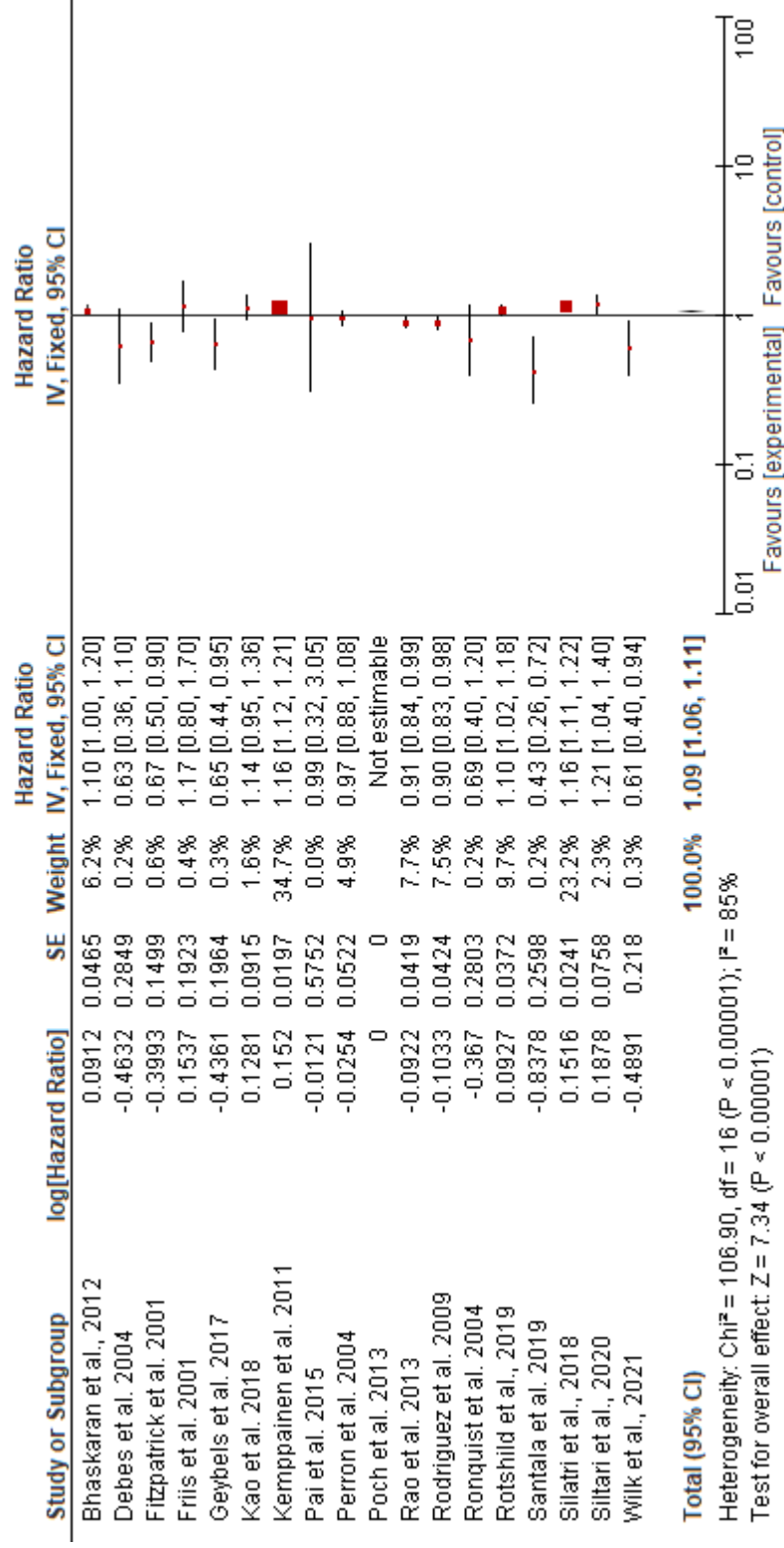

Supplement: Supplementary file 2 — Supplementary Material 2 [file 12885_2024_12218_MOESM2_ESM.pdf]
