# Supplementary material for: Role of antihypertensive medicines in prostate cancer: a systematic review
Source: BMC Cancer. 2024 Apr 29;24:542. doi: 10.1186/s12885-024-12218-5 (PMC11059764; doi:10.1186/s12885-024-12218-5)
Supplement: Supplementary file 4 — Supplementary Material 4 [file 12885_2024_12218_MOESM4_ESM.doc]

Supplementary Table 2

| Authors, Year, Country | Was the research question or objective in this paper clearly stated and appropriate? | Was the study population clearly specified and defined? | Did the authors include a sample size justification? | Were controls selected or recruited from the same or similar population that gave rise to the cases (including the same timeframe)? | Were the definitions, inclusion and exclusion criteria, algorithms or processes used to identify or select cases and controls valid, reliable, and implemented consistently across all study participants? | Were the cases clearly defined and differentiated from controls? | If less than 100 percent of eligible cases and/or controls were selected for the study, were the cases and/or controls randomly selected from those eligible? | Was there use of concurrent controls? | Were the investigators able to confirm that the exposure/risk occurred prior to the development of the condition or event that defined a participant as a case? | Were the measures of exposure/risk clearly defined, valid, reliable, and implemented consistently (including the same time period) across all study participants? | Were the assessors of exposure/risk blinded to the case or control status of participants? | Were key potential confounding variables measured and adjusted statistically in the analyses? If matching was used, did the investigators account for matching during study analysis? | Total |
| --- | --- | --- | --- | --- | --- | --- | --- | --- | --- | --- | --- | --- | --- |
| Rotshild et al., 2019  Isreal | 1 | 1 | 0 | 1 | 1 | 1 | 1 | 1 | 1 | 1 | 0 | 1 | 10 |
| Geybels et al. 2017  USA | 1 | 1 | 0 | 1 | 1 | 1 | 1 | 1 | 1 | 1 | 0 | 1 | 10 |
| Ronquist et al. 2004  Sweden | 1 | 1 | 1 | 1 | 1 | 1 | 1 | 1 | 1 | 1 | 0 | 1 | 11 |
| Perron et al. 2004  Canada | 1 | 1 | 1 | 1 | 1 | 1 | 1 | 1 | 1 | 1 | 0 | 1 | 11 |
| Kemppainen et al. 2011  Finland | 1 | 1 | 1 | 1 | 1 | 1 | 1 | 1 | 1 | 1 | 0 | 0 | 10 |
| Kao et al. 2018  China | 1 | 1 | 0 | 1 | 1 | 1 | 1 | 1 | 1 | 1 | 0 | 1 | 10 |
| Pai et al. 2015  Taiwan | 1 | 1 | 1 | 1 | 1 | 1 | 1 | 1 | 1 | 1 | 0 | 1 | 11 |
